# Effects of Age and Dietary Factors on the Blood Beta-Hydroxybutyric Acid, Metabolites, Immunoglobulins, and Hormones of Goats

**DOI:** 10.3389/fvets.2021.793427

**Published:** 2022-02-09

**Authors:** Mahmoud M. Abdelsattar, Einar Vargas-Bello-Pérez, Yimin Zhuang, Yuze Fu, Naifeng Zhang

**Affiliations:** ^1^Institute of Feed Research of Chinese Academy of Agricultural Sciences, Key Laboratory of Feed Biotechnology of the Ministry of Agriculture and Rural Affairs, Beijing, China; ^2^Department of Animal and Poultry Production, Faculty of Agriculture, South Valley University, Qena, Egypt; ^3^Department of Veterinary and Animal Sciences, Faculty of Health and Medical Sciences, University of Copenhagen, Frederiksberg, Denmark

**Keywords:** beta-hydroxybutyric acid, age, weaning, blood chemical composition, immunoglobulins

## Abstract

The study was aimed to examine the effects of age and dietary beta-hydroxybutyric acid (BHBA) on blood BHBA and blood health indicators in goat kids. Thirty male goats of five ages (1, 2, 3, 6, and 12 months old) were selected for blood sampling to determine the influence of age. Another 64 goat kids (half males and half females) were weaned at 1 month old and fed with starter diets with control, low, medium, and high BHBA doses (0, 3, 6, and 9 g/animal/day, respectively). Six goats per treatment were selected for blood analysis at 2 and 3 months of age. There were significant effects (*p* < 0.01) of ages on the blood parameters of goat kids. The 6- and 12-month-old goats showed significantly lower blood total protein, globulin, BHBA, IgA, and IgM concentrations than did young goats, while they had a higher albumin-to-globulin ratio than young goats. The blood glucose decreased (*p* < 0.01) and IgG increased over time (*p* < 0.01). In blood, growth hormone (GH) and insulin-like growth factor I (IGF-I) were lower (*p* < 0.01) at 1- and 3-month-old goats than 12-month-old goats. The high dietary BHBA improved (*p* < 0.05) the ratio of albumin to globulin of 2-month-old kids compared with control. The blood GH and IGF-I were lower (*p* < 0.01) in the medium BHBA dose at 2 months of age than control. These results suggested that age greatly impacted blood composition, especially around weaning, and dietary BHBA showed beneficial regulating effects on blood total protein level in young goats.

## Introduction

The blood metabolites can reflect the nutritional state of animals (1), which is associated with performance and health status. For example, globulin (GLB) and immunoglobulins (Ig) are used to indicate immune status (2). Both the blood proteins and immune status improvement are associated with the secretion of growth hormone (GH) (2), which stimulates the synthesis of insulin-like growth factor I (IGF-I) in the liver (3). As a growth-promoting hormone, IGF-I is considered to regulate tissue growth and differentiation (4). At the same time, insulin (INS) is synthesized in the pancreatic β-cells (5). The anabolic role of plasma IGF-I and INS are stimulating the uptake of amino acids and glucose (GLU) (6–8). Therefore, blood metabolites, immunoglobulins, and hormones in goats are interconnected and deserve extensive investigation.

Goats (Caprinae) start as monogastric and become small ruminants within 2 months of age (9). The blood indexes of goats can be affected by the dynamics and processes of weaning and the maturation of the rumen, liver, and immune system during the transition from pre-ruminant to ruminant (10). The volatile fatty acids (VFA), especially the butyric acid produced by the rumen fermentation of solid feed, were considered the main stimulators of rumen development (11–13). Butyrate absorbed by rumen appears as beta-hydroxybutyric acid (BHBA) and acetoacetate in portal blood (14), suggesting that BHBA may be a further stimulator of rumen development. It has been indicated that blood BHBA and ketogenesis processes could be affected by diet, stress, or age (15). Indeed, blood BHBA of calves increased with the increase of starter intake and was higher at 7 weeks than at 6 weeks (15). In addition, BHBA has been used to indicate subclinical ketosis in dairy cattle due to its positive correlation with milk BHBA (16), which suggests a relationship between BHBA and health indicators. However, the relationship between blood BHBA and gut development over time, and between dietary BHBA and blood BHBA has not been described in goats. This study hypothesized that blood BHBA and health indicators of goats could vary with age, especially around weaning, and the dietary BHBA could regulate these changes due to its role in rumen development. Therefore, the study objectives were (1) to examine the effect of age on blood BHBA and other metabolites, Ig, and hormones and (2) to evaluate the impact of BHBA-based diets on blood metabolites, Ig, and hormones in growing goats.

## Materials and Methods

This study was conducted between June 10 and August 10, 2020, at Haimen goat farm, Jiangsu, China (latitude, 31°53′N; longitude 121°09′E). The experimental work was performed following the guidelines approved by the Animal Ethics Committee of the Chinese Academy of Agricultural Sciences (protocol number: AEC-CAAS-20200605; approval date: June 3, 2020).

### Animals and Experimental Design

The Yangtze River Delta white goat is a unique indigenous Chinese breed that is widely used for meat and high-quality bush hair. The Yangtze River Delta white goat was included in two research experiments. Experiment 1 was designed to determine the effect of age on blood BHBA and blood health indicators. Thirty male goats were randomly selected from the farm at different ages (1, 2, 3, 6, and 12 months) for blood collection. Experiment 2 was designed to determine the effect of age, dietary BHBA, and its interaction on blood BHBA and blood health indicators. For this goal, 64 goats at 1 month old were separated from their dams and randomly assigned to a 60-day feeding trial. Kids were reared in one shed, and one male and one female were assigned to one pen (2 kids/pen; 2 m × 2 m) based on body weight (5.14 ± 0.13 kg birth weight; mean ± SEM). In this way, the goats were randomly assigned to 1 of 4 groups in 32 pens: control, 3 g/day of BHBA (low dose), 6 g/day of BHBA (medium dose), and 9 g/day of BHBA (high dose). At 2 and 3 months old, six goats were per treatment were selected to study the short- and long-term effects of dietary BHBA supplementation on blood composition. The body weight of the abovementioned selected goat kids was recorded before the blood sampling. After 3 months of age, the kids were transferred into wider pens (20 kids per pen; 4 m × 4 m).

### Diets and Feeding Management

Diets for goat kids were formulated to meet growth requirements according to the National Research Council (NRC) nutrient specification as previously described (17). Goats had free access to water. Weaned goats were fed with milk replacer thrice as 2% of body weight and starter *ad libitum* twice a day at 0800 and 1400 h (Table 1). After 2 months, the milk replacer was removed, and kids were fed with only starter *ad libitum*. After 3 months of age, the kids were offered concentrate *ad libitum* and corn and soybean straw. For experiment 2, the goats were fed with the abovementioned control diet, or the diets were supplemented with different doses of BHBA. The chemical composition and ingredients of the milk replacer and concentrate diets are shown in Table 1.

**Table 1:** Ingredients and chemical composition (dry matter basis) of the concentrate diet and milk replacer.

**Items**	**Concentrate diet**	**Milk replacer**
**Ingredients, %**
Corn	50.0	–
Soybean meal	25.0	–
Bran	10.0	–
Premix^a^	2.50	–
Salt	0.50	–
Sodium bicarbonate	1.00	–
Calcium bicarbonate	4.00	–
Corn husk	15.0	–
**Chemical composition**
Dry matter, %	91.2	95.5
Metabolizable energy^b^, Mcal/kg	3.20	4.63
Ether extract, %	3.73	16.0
Crude protein, %	19.4	25.5
Calcium, %	0.95	1.02
Phosphorus, %	0.70	0.66

a *The premix provided the followings per kg of diet: VA, 12,000 IU; VD, 2,000 IU; VE, 30 IU; Cu, 12 mg; Fe, 64 mg; Mn, 56 mg; Zn, 60 mg; I, 1.2 mg; Se, 0.4 mg; Co, 0.4 mg; Ca, 3.2 g; P, 1.2 g; NaCl, 6.4 g*.

b *The metabolizable energy was calculated by the equations from NRC (18)*.

### Blood Collection and Analysis

Blood samples obtained from jugular venipuncture were collected in 10-ml Vacutainer tubes without anticoagulants. After centrifugation at 3,000 rpm for 10 min, the supernatant was transferred to 1.5-ml Eppendorf tubes and stored at −20°C until further analysis. Blood biochemical analyses were performed at the laboratory of Beijing Jinhai Keyu Biotechnology Development Co., Ltd., Beijing, China. Determinations of biochemical indices as blood total protein (TP), albumin (ALB), and GLU were performed using a KHB-1280 automatic biochemical analyzer (Kehua Biological Engineering Co., Ltd., Shanghai, China). In addition, blood IgA, IgG, and IgM were quantified colorimetrically using a KHB-1280 Automatic Biochemical Analyzer Kehua Biological Engineering Co., Ltd., Shanghai, China). The blood GLB was determined by the difference between TP and ALB concentrations. The ratio between ALB and GLB (A/G) was calculated. The blood INS, GH, IGF-I, and BHBA concentrations were determined using an ELISA commercial kit (Beijing Jinhai Keyu Biotechnology Development Co., Ltd.), following the manufacturer's instructions, and an ST-360 microplate reader (Kehua Biological Engineering Co., Ltd., Shanghai, China). No cross-reactions were observed with other soluble structural analogs. All coefficients of variation for intra- and inter-assay were <10%. The assay sensitivity was <0.1 mIU/L for INS, 0.1 ng/ml for GH, 1.0 ng/ml for IGF-I, and 0.1 mmol/L for BHBA.

### Statistics

Data were tested for normality distribution (Shapiro–Wilk test) before the statistical analysis. The data showed a normal distribution (*p* > 0.05). The data for goats at different ages (1, 2, 3, 6, and 12 months of age) were analyzed by the one-way ANOVA of SAS (SAS Enterprise Guide 5.1, SAS Institute Inc., Cary, NC, USA). The data of goats fed with BHBA were analyzed by the two-way ANOVA of SAS that included the fixed effects of age (2 and 3 months) and dietary BHBA (control, low, medium, and high doses of 0, 3, 6, and 9 g/animal/day of BHBA, respectively) and the interaction between age and BHBA dose. Data were presented as arithmetic means and standard errors. Significant differences between groups were determined by Tukey's *post-hoc* test. Significance was set at *p* < 0.05. In addition, Pearson's correlation coefficient between the blood parameters was estimated in R studio (v. 1.3.1073), in which a probability of *p* < 0.05 indicated the significant differences.

## Results

### Experiment 1

Animal age effects on blood parameters are shown in Table 2. Low blood TP and GLB (*p* < 0.01) were observed at 6 and 12 months old compared with younger goats, while the TP values of 12-month-old goats were higher than those of 6-month-old goats. Meanwhile, the blood ALB did not show significant differences over time. However, the A/G ratios of both 6- and 12-month-old kids were higher (*p* < 0.01) than those of the younger kids. In contrast, the blood GLU concentrations at 1 month old were higher (*p* < 0.01) than in the following months. In addition, the blood BHBA concentrations were higher in younger goats (1–3 months; *p* < 0.01) than senior kids (6–12 months).

**Table 2:** Blood parameters of goats from 1 to 12 months of age (*n* = 6 per group).

**Parameters**	**Age**					**SEM**	***p*-value**
	**1 months**	**2 months**	**3 months**	**6 months**	**12 months**		
**Metabolites**
TP, g/L	68.9^a^	70.7^a^	69.0^a^	58.8^c^	63.6^b^	1.02	<0.01
ALB, g/L	34.7	33.1	33.0	33.1	33.8	0.31	0.37
GLB, g/L	34.3^ab^	37.6^a^	35.1^a^	25.6^c^	29.7^bc^	0.96	<0.01
A/G, %	1.01^b^	0.88^b^	0.92^b^	1.29^a^	1.17^a^	0.03	<0.01
GLU, mmol/L	5.36^a^	3.85^b^	3.33^b^	3.73^b^	3.51^b^	0.15	<0.01
BHBA, mmol/L	1.00^a^	0.90^a^	0.96^a^	0.53^b^	0.51^b^	0.05	<0.01
**Immunoglobulins**
IgA, g/L	0.92^a^	0.84^ab^	0.79^b^	0.42^c^	0.52^c^	0.04	<0.01
IgG, g/L	9.75^c^	10.4^c^	11.0^c^	17.6^b^	20.5^a^	0.89	<0.01
IgM, g/L	2.31^a^	2.64^a^	2.43^a^	0.98^b^	1.32^b^	0.14	<0.01
**Hormones**
INS, mIU/L	20.8	14.1	13.1	16.2	22.5	1.31	0.08
GH, ng/ml	3.42^bc^	3.85^ab^	3.09^c^	3.44^bc^	4.09^a^	0.10	0.01
IGF-I, ng/ml	53.1^c^	63.8^abc^	54.6^bc^	64.9^ab^	71.9^a^	1.68	<0.01

*TP, total protein; ALB, albumin; GLB, globulin; A/G, ratio between albumin and globulin; GLU, glucose; BHBA, beta-hydroxybutyric acid; INS, insulin; GH, growth hormone; IGF-I, insulin-like growth factor I*.

a, b, c *Means with different superscripts in each row differ significantly (p < 0.05)*.

Furthermore, the blood IgA concentrations were lower (*p* < 0.01) at 3 months than at 1 month. Then, the IgA concentrations were also lower at 6 and 12 months than younger goats. Low blood levels of IgM (*p* < 0.01) were observed within 6 to 12 months of age. However, the blood IgG increased (*p* < 0.01) over time, and that of 6-month-old kids were higher than that of younger goat. In addition, the 12-month-old kids showed the highest IgG concentrations.

In addition, blood GH was lower (*p* < 0.01) at 1 month than 12 months, and it was lower at 3 months than at both 2 and 12 months of age. In addition, the blood IGF-I values were lower at 1-month-old goats than 6- and 12-month-old goats (*p* < 0.01), and the 3-month-old goats showed lower (*p* < 0.01) values than the 12-month-old goats. However, the blood INS did not show significant differences over time.

**Figure 1:**
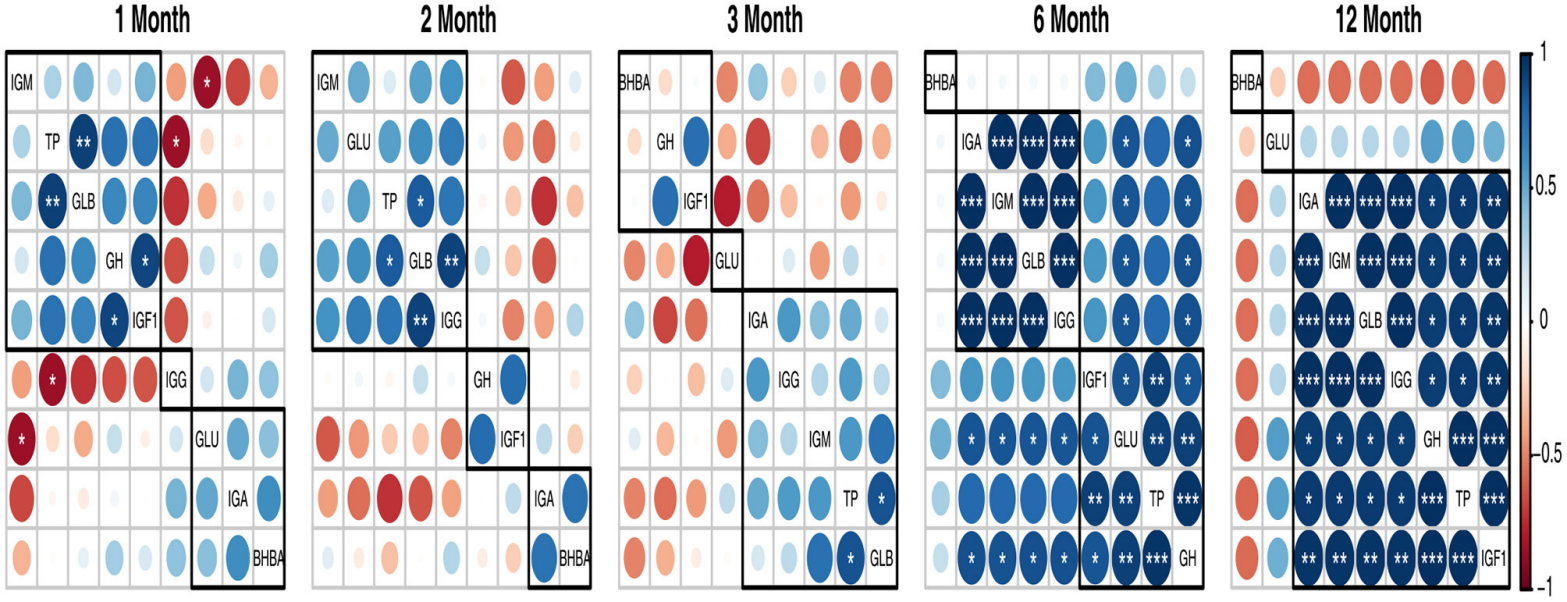
A heatmap illustrating Pearson's correlation coefficients between blood parameters in goats from 1 to 12 months of age. Significance levels: *p < 0.05, **p < 0.01, and ***p < 0.001.

In Figure 1, the blood TP, GLB, BHBA, IgA, and IgM were positively correlated (*p* < 0.01), but they were negatively correlated (*p* < 0.01) with A/G ratio and IgG. ALB had a positive correlation (*p* < 0.01) with TP and GLU. In addition, the blood GLU was positively correlated with IgA (*p* < 0.01), TP, and BHBA (*p* < 0.05), but it was negatively correlated with IgG (*p* < 0.05). In addition, INS was positively correlated (*p* < 0.05) with the A/G ratio but negatively correlated (*p* < 0.05) with GLB and IgM. Furthermore, the GH was positively correlated with IgG (*p* < 0.05) and IGF-I (*p* < 0.01). Moreover, blood IGF-I had a positive correlation with IgG (*p* < 0.01) and a negative correlation with IgA, IgM (*p* < 0.05), and BHBA (*p* < 0.01).

### Experiment 2

The effect of dietary BHBA on the blood parameters of goat kids is shown in Table 3. High dietary BHBA dose increased blood ALB (*p* < 0.05) compared with that of the control group. Moreover, both low and high BHBA doses showed a higher A/G ratio (*p* < 0.01) in comparison with that of the control group. The blood GLU was lower (*p* < 0.01) in the medium BHBA dose than the low and high BHBA doses.

**Table 3:** Effect of dietary BHBA on blood parameters of goat kids (*n* = 6 per group).

**Parameters**	**Age**	**Treatments**					**SEM**	***p*** **-value**		
		**Control**	**Low**	**Medium**	**High**	**Mean**		**Age**	**Treatment**	**Interaction**
**Metabolites**
TP, g/L	2 months	70.7	69.8	69.1	69.9	69.9	0.80	0.33	0.48	0.65
	3 months	69.0	69.4	68.7	70.1	69.3				
	Mean	69.9	69.6	68.9	70.0					
ALB, g/L	2 months	33.1	34.0	33.8	34.9	33.9	0.51	0.30	0.03	0.84
	3 months	33.0	34.0	33.1	34.2	33.6				
	Mean	33.0^B^	34.0^AB^	33.4^AB^	34.5^A^					
GLB, g/L	2 months	37.6^a^	35.7^b^	35.3^b^	35.0^b^	35.9	0.40	0.26	0.10	<0.01
	3 months	35.1^b^	35.3^b^	35.9^ab^	35.9^ab^	35.6				
	Mean	36.4	35.5	35.6	35.5					
A/G, %	2 months	0.88^b^	0.95^ab^	0.96^ab^	1.00^a^	0.95	0.02	0.33	0.01	0.04
	3 months	0.92^ab^	0.96^ab^	0.90^b^	0.95^ab^	0.93				
	Mean	0.90^B^	0.96^A^	0.93^AB^	0.98^A^					
GLU, mmol/L	2 months	3.85	4.06	3.92	4.41	4.06^A^	0.18	<0.01	0.01	0.08
	3 months	3.33	3.71	2.78	3.33	3.29^b^				
	Mean	3.59^AB^	3.88^A^	3.35^B^	3.87^A^					
BHBA, mmol/L	2 months	0.90	0.92	0.98	1.00	0.95	0.06	0.60	0.89	0.55
	3 months	0.96	1.02	0.98	0.94	0.97				
	Mean	0.93	0.96	0.98	0.97					
**Immunoglobulins**
IgA, g/L	2 months	0.84	0.79	0.88	0.88	0.85	0.04	0.72	0.34	0.20
	3 months	0.79	0.88	0.83	0.87	0.84				
	Mean	0.81	0.83	0.86	0.88					
IgG, g/L	2 months	10.4	9.38	9.69	9.84	9.82	0.74	0.14	0.68	0.81
	3 months	11.0	11.0	9.84	10.7	10.6				
	Mean	10.7	10.1	9.76	10.3					
IgM, g/L	2 months	2.64	2.61	2.77	2.41	2.61	0.19	0.53	0.63	0.88
	3 months	2.43	2.56	2.61	2.48	2.52				
	Mean	2.53	2.58	2.69	2.44					
**Hormones**
INS, mIU/L	2 months	14.1	17.8	16.9	18.0	16.7	2.05	0.09	0.42	0.90
	3 months	13.1	15.6	12.9	14.9	14.1				
	Mean	13.6	16.8	14.9	16.45					
GH, ng/ml	2 months	3.85^a^	3.79^ab^	3.19^bc^	3.66^abc^	3.62^A^	0.14	<0.01	0.38	0.01
	3 months	3.09^c^	3.08^c^	3.30^abc^	3.21^bc^	3.17^B^				
	Mean	3.47	3.44	3.24	3.43					
IGF-I, ng/ml	2 months	63.8^ab^	68.7^a^	56.9^bcd^	62.2^abc^	62.9^A^	1.96	<0.01	0.22	0.01
	3 months	54.6^cd^	49.8^d^	54.4^cd^	55.6^bcd^	53.6^b^				
	Mean	59.2	59.2	55.7	58.9					

*TP, total protein; ALB, albumin; GLB, globulin; A/G, ratio between albumin and globulin; GLU, glucose; BHBA, beta-hydroxybutyric acid; INS, insulin; GH, growth hormone; IGF-I, insulin-like growth factor I*.

A, B *Different superscripts in each row of mean (doses) and column of mean (age) differ significantly (p < 0.05)*.

a, b, c, d *Different superscripts in the interaction of BHBA dose and age indicate significant differences (p < 0.05). The control group was fed without dietary supplementation; and the low-, medium-, and high-dose groups were fed with 2, 6, and 9 g/animal/day of BHBA, respectively*.

Blood GLU, GH, and IGF-I decreased (*p* < 0.01) over time. Blood GH and IGF-I showed a significant interaction between diet and age (*p* < 0.01), and the control group had higher GH than the medium BHBA dose group at 2 months. In addition, the low dose of BHBA showed elevated blood IGF-I than the medium BHBA dose at 2 months. However, the blood concentrations of TP, GLB, BHBA, INS, and Ig were not affected by dietary BHBA and age.

**Figure 2:**
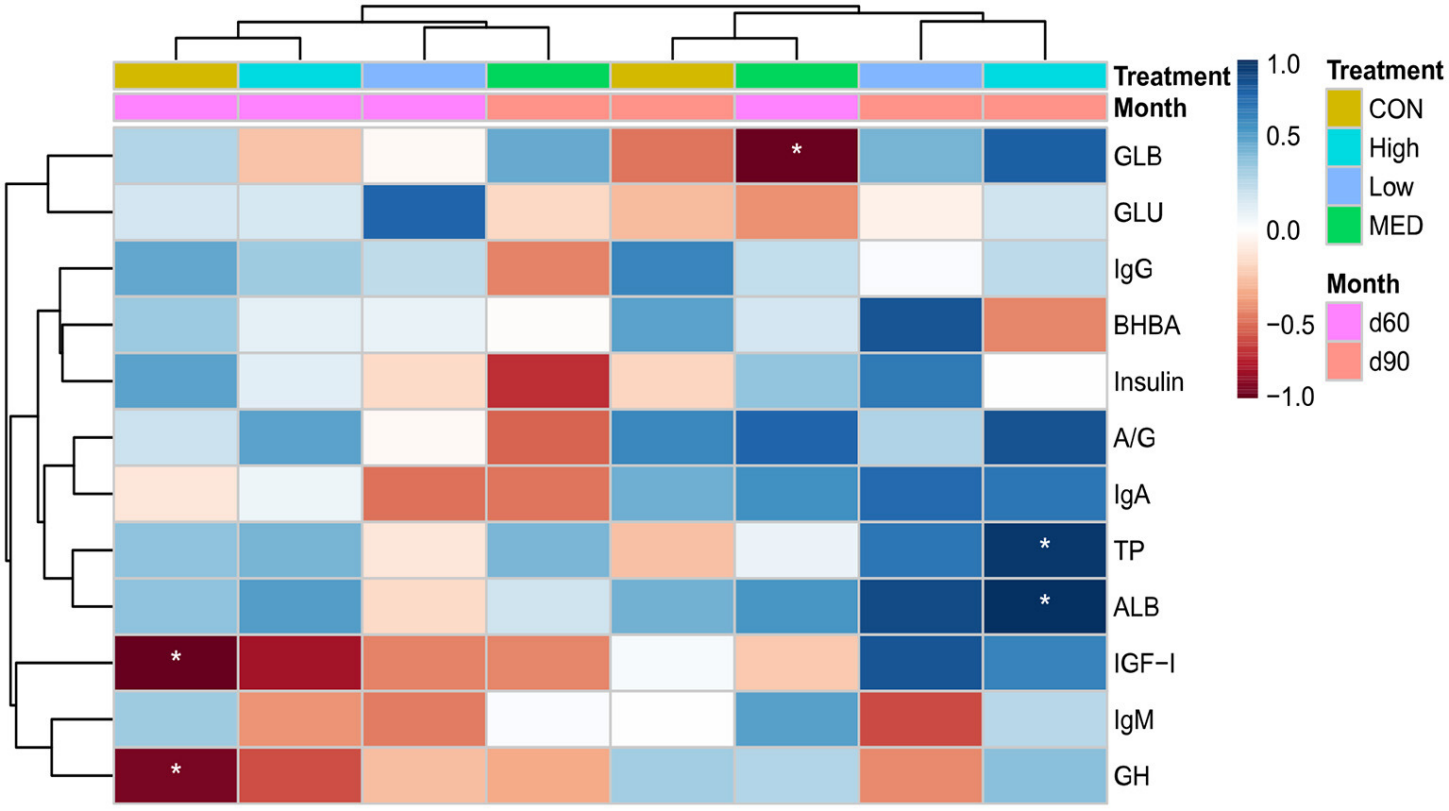
A heatmap illustrating Pearson's correlation between body weight and blood parameters of goat kids supplemented with different concentrations of BHBA. Significance levels: *p < 0.05. The control group was fed without dietary supplementation; and the low-, medium-, and high-dose groups were fed with 2, 6, and 9 g/animal/day of BHBA, respectively. BHBA, beta-hydroxybutyric acid.

In the control group at 2 months, body weight had a negative relationship (*p* < 0.05) with GH and IGF-I (Figure 2). In addition, the body weight was negatively correlated (*p* < 0.05) with blood GLB in medium BHBA dose during 2 months of age. However, the blood TP and ALB were positively (*p* < 0.05) correlated with body weight in the high BHBA dose group at 3 months of age.

## Discussion

### Experiment 1

Blood proteins are indicators for neonates' immunity and growth (19). The optimal blood protein levels could reduce the mortality rates in young animals (20). In this study, the blood TP and GLB concentrations were higher in 1–3-month-old than 6-month-old goats, but ALB was not affected by age. The weaning stress of goat kids could influence the immune status (21) by increasing blood GLB concentration (22). However, these parameters showed a subsequent increase at 12 months than at 6 months of age. This should be related to the age growth and physiological maturity of kids. Indeed, the blood TP, ALB, and GLB concentration of kids increased with age (23). The enhanced protein intake due to physiological maturity caused extensive degradation of dietary protein and metabolism of absorbed amino acids, which eventually led to the possible increase of blood protein level (24).

After 1 month of age, blood GLU in goats decreased over time. Blood GLU concentration decreased (*p* < 0.001) with the increasing age of kids (25). Interestingly, blood GLU concentrations in Saharan goats were high 1 week after parturition and then significantly decreased at 8, 11, and 12 weeks of lactation due to the negative energy balance (26). Goat kids had significantly high blood GLU in the non-ruminant phase (10), in which colostrum and milk keep high contents of body GLU (27), while the decrease of milk and the developed rumen caused a reduction in blood GLU (28, 29). Rumen development caused low GLU and high starter intake over time (30). The developed rumen replaces GLU with rumen fermentation products as the main energy source (31, 32). Both GLU and butyrate at birth have the same oxidation rate, while butyrate metabolism to ketones increases six-fold at weaning (33). Early weaning could enhance growth by increasing VFA (34). Despite the beneficial effects of BHBA for rumen development, the higher blood ketones bodies (including BHBA) indicate energy deficiency and are considered an indicator of subclinical ketosis (35). In this study, the blood BHBA was lower in goats at 6 and 12 months than younger goats, indicating the health status of goat kids and the better nutrition state. In this study, blood BHBA had a positive relationship with blood GLU, which is inconsistent with the results of Zarrin (36), who demonstrated that the infusion of BHBA increased the blood INS and decreased the blood GLU in Holstein dairy cows. Further research is still needed to clarify the relation between these metabolites.

IgG is the most preponderant blood antibody compared with IgA and IgM (37). In this study, IgG contents increased over time, which is consistent with the results of Piccione (38), who found a low level of serum IgG in newborn kids compared with the mothers. In addition, the IgG showed a positive correlation with the days of life of newborns (38). The rising concentration of IgG can be linked to the maturation of the immune system caused by antigenic stimulation (39). However, blood IgM and IgA concentrations were high in younger goats and decreased over time, suggesting that both IgA and IgM were the first antibodies to prevent weaning stress. The IgA also has local activity in the gut against diarrhea. The increase in blood IgG and the decrease in blood IgA and IgM over time in goat kids suggest the imbalance of the internal environment and increased immune stimulation (40).

This study observed a positive correlation between blood proteins (TP and GLB) and immunoglobulins (IgA and IgM). Similarly, dietary protein promotes the absorption of immunoglobulins and IGF-I in calves fed with colostrum (41). In addition, a ewe protein diet in late pregnancy increased the absorption efficiency of IgG (42). However, the uptake of these large immunomolecules decreases during the first days of life due to gut closure (43). Consequently, the increase of TP, GLB, IgA, and IgM in this study could be due to weaning stress more than the improvement of absorption efficiency.

A significant reduction was observed in GH and IGF-I at 1- and 3-month-old goats than 12-month-old goats. At birth and after weaning, newborn animals are probably exposed to various morphological, metabolic, and physiological changes that can affect blood metabolites and enzymes (44). Thus, weaning decreased blood IGF-I (22, 45) due to reduced circulating IGF-I during poor nutrition (46). In lambs fed with milk, Sun (47) showed that milk was rich in IGF-I and had higher blood IGF-I than lambs fed with milk plus starter diets. Subsequently, INS, GH, and IGF-I increased over time, possibly due to improved nutrition and rumen development. Energy and protein intake are considered the main factors affecting plasma IGF-I concentration (48, 49). In this regard, Shen et al. (50) indicated that young goats fed with high-energy diets had higher plasma IGF-I concentrations and expression of IGF-I receptors in the rumen epithelium than goats fed with low-energy diets. In addition, Hua et al. (51) reported that exogenous bovine GH increased (*p* < 0.01) the plasma levels of IGF-I in the fed with rather than fasted sheep; in addition, fasting decreased the levels of IGF-I.

### Experiment 2

Blood proteins are used as indicators of gut metabolic function and health status (52, 53). In this study, the group with the high dose of BHBA had an elevated blood ALB and A/G ratio as compared with those of the control group. Furthermore, the blood TP and ALB were positively correlated with body weight in the high BHBA dose. Such beneficial effects could be related to stimulated ruminant digestive system particularly for digestion of protein and fat (54). BHBA is linked with the high intake of solid feed, microbial communities, and rumen development of fermentation and absorption processes (15, 55). Moreover, the solid feed intake can increase VFA concentrations, alter the rumen microbiome (56), and improve rumen epithelium metabolic function (57). The development of rumen epithelium depends on VFA absorption, transportation, and metabolism (58). Accordingly, the supply of BHBA in this study can promote rumen functioning, and this was reflected by the improvement of blood proteins and animal health status. On the other hand, diets linked with low rumen VFA, such as milk replacer, showed slower development of the rumen and the small intestine (59). The lower blood ALB found in the control group could be related to the interruption of suckling and the incomplete digestive system to degrade solid diets (53, 60).

However, health risks have been reported in livestock due to elevated blood ketones (35). The feed intake of the medium BHBA group decreased while the high BHBA group did not show a similar response due to the adaptation behavior (61). Only medium BHBA dose decreased blood GLU (overall) and IGF-I (only at 2 months) compared with the low BHBA dose. Herrick (62) showed that infusing butyrate increases blood butyrate and BHBA concentrations and decreases GLU. In this study, blood BHBA showed a numerical increase in kids receiving BHBA than the control group at 2 months of age. Several studies showed a positive relationship between blood BHBA and the starter feed intake (55, 63). However, other studies reported the importance of the age factor than the starter feed on BHBA production in rumen epithelium (64, 65), which could be the most likely cause of nonsignificant effect of dietary BHBA on blood ketones after weaning.

The GH and IGF-I were negatively correlated with body weight in the control group at 2 months. The abrupt alteration in the diet caused by weaning may be associated with a period of decreased or stagnant development (66, 67). However, the control group showed high GH levels only at 2 months, which could be due to dehydration due to the abrupt diet change. In another study, frequent diarrhea, mortality, and body weight loss are the possible signs of stress within 2 weeks after weaning (68). On the other hand, the high GH could be due to under-nutrition status after weaning (45).

## Conclusions

According to blood parameters, this longitudinal study showed that the blood concentrations of TP, GLB, IgA, IgM, and BHBA were higher in young kids before and after weaning (1–3 months) than in older goats (6 and 12 months). However, the A/G ratio, GH, and IGF-I were lower in young animals than adult animals. The dietary BHBA supplementation did not affect blood BHBA, but at high BHBA dosing, it elicited a higher blood ALB and A/G ratio than the control group. Future research is still needed to investigate the usefulness of BHBA in improving rumen development growth performance and health status in young ruminants.

## Data Availability Statement

The raw data supporting the conclusions of this article will be made available by the authors, without undue reservation.

## Ethics Statement

The animal study was approved according to the Animal Ethics Committee of the Chinese Academy of Agricultural Sciences (Protocol number: AEC-CAAS-20200605).

## Author Contributions

MA contributed to the methodology, software, writing—original draft, and writing—review and editing. EV-B-P contributed to the validation and writing—review and editing. YZ and YF contributed to the methodology. NZ contributed to the investigation, project administration, resources, supervision, validation, visualization, and writing—review and editing. All authors contributed to the article and approved the submitted version.

## Funding

This study was funded by grants from the National Natural Science Foundation of China (31872385) and the National Key R&D Program Projects (2018YFD0501902).

## Conflict of Interest

The authors declare that the research was conducted in the absence of any commercial or financial relationships that could be construed as a potential conflict of interest.

## Publisher's Note

All claims expressed in this article are solely those of the authors and do not necessarily represent those of their affiliated organizations, or those of the publisher, the editors and the reviewers. Any product that may be evaluated in this article, or claim that may be made by its manufacturer, is not guaranteed or endorsed by the publisher.
